# Organising health research systems as a key to improving health: the World Health Report 2013 and how to make further progress

**DOI:** 10.1186/1478-4505-11-47

**Published:** 2013-12-17

**Authors:** Stephen R Hanney, Miguel A González-Block

**Affiliations:** 1 Health Economics Research Group, Kingston Lane, Brunel University, Uxbridge UB8 3PH, UK; 2 Center for Health Systems Research, National Institute of Public Health, Av. Universidad 655, Cuernavaca, Morelos 52608, Mexico

## Abstract

The World Health Report 2013 provides a major boost to the health research community and, in particular, to those who believe that health research will make its greatest impact on improving health when it is organised through a systems approach. The World Health Report 2013, *Research for Universal Health Coverage*, starts with three key messages. Firstly, that universal health coverage, with full access to high-quality services, needs research evidence if it is to be achieved; second, all nations should conduct and use research; and finally, the report states that systems are needed to develop national research agendas, to raise funds, to strengthen research capacity, and to make effective use of research findings. Each of these themes is elaborated in the report and supported by extensive references.

In this editorial, we first outline the key messages from the World Health Report 2013 and highlight the contributions made by papers from our journal, *Health Research Policy and Systems*. In addition, we discuss very recent papers that advance some issues even further. In particular, we consider new evidence both on how to achieve financial protection for those who use health services, and on whether healthcare professionals and organisations who engage in research provide an improved healthcare performance. Finally, we propose additional perspectives that add to the impressive body of evidence and analyses presented in the report. Specifically, we suggest that considering the needs of various stakeholders, as attempted in the UK, in parallel with analysing how to fulfil essential functions, should boost the prospects of successfully building and strengthening health research systems. This is important because research is vital for achieving universal health coverage, and consequently for improving the health of millions of people.

## Background

The keenly anticipated World Health Report (WHR) 2013, *Research for Universal Health Coverage*[1], has given a major boost to the health research community and, in particular, to those who believe that health research will make its greatest impact on improving health when it is organised through a systems approach. The WHR 2013 starts with three key messages that are well matched to the mission of our journal, *Health Research Policy and Systems* (*HARPS*).

The first message is that ‘Universal health coverage, with full access to high-quality services… cannot be achieved without evidence from research.’ Second, ‘All nations should be producers of research as well as consumers.’ Finally, and from our perspective most interestingly, the report states that ‘To make the best use of limited resources, systems are needed to develop national research agendas, to raise funds, to strengthen research capacity, and to make appropriate and effective use of research findings’ [1], p. xi. Each of these themes is elaborated in the report and supported by extensive references. We are pleased to note that several articles published in *HARPS* are cited as key references in the section on building health research systems. Indeed, we have striven to ensure the promotion of this field of study in *HARPS*, as attested by our editorial in 2008 [2] when we presented a series of papers and stated that ‘What unites these forthcoming papers in *HARPS* is the focus on analysis of the ways health research systems can best be organised to improve health care. This is central to the mission of *HARPS*’ [2], p. 2.

In this editorial, we outline the key messages from the WHR 2013 [1] and highlight the contributions made by papers from *HARPS*. In addition, we draw attention to recent papers, in *HARPS* or elsewhere, that further advance aspects of the evidence on some of the issues covered. Finally, we propose additional perspectives that might add to the impressive body of evidence and analysis presented in the report. These perspectives should boost the prospects of successfully building health research systems, which is of major importance because, in the words of the WHR, ‘research is vital for achieving universal health coverage, and consequently for improving the health of all people around the world’ [1], p. 137.

### Research to achieve universal health coverage

In relation to the first message, the WHR 2013 [1] emphasises the commitment of the World Health Organization (WHO) to achieve the two facets of universal health coverage (UHC): ‘the provision of, and access to, high-quality health services; and financial risk protection for people who need to use these services’ [1], p. xi. The report sets out both how the 2005 commitment by all WHO Member States to achieve UHC [3] launched an agenda for research, and how research is the means by which promising ideas are turned into practical solutions for improving health.

Evidence about how best to secure financial access continues to accumulate. A recent series of projects funded by the Alliance for Health Policy and Systems Research, and published in *HARPS*[4-10], aims to identify and support the use of evidence from seven low- and middle-income countries on factors facilitating or hindering progress towards universal financial protection. This evidence is drawn together by McIntyre et al., and underlines ‘the critical role of high-level political leadership in pursuing UHC policies and citizen support in sustaining these policies’ [11], p. 1.

The case studies from Costa Rica [4] and Thailand [5], the two upper-middle-income countries in the series, describe, according McIntyre et al. [11], remarkable progress towards UHC. Nevertheless, ‘both countries embarked on ambitious programs of expanding coverage when still considered to be lower-middle or lower-income countries’ [11], p. 2. The papers from Georgia [6], India [7], and Malawi [8] describe recent initiatives aimed at extending health insurance coverage to the poorest populations through the use of government revenue. In the case of Malawi, the scheme involves direct payments to faith-based facilities, with a particular focus on maternal and neo-natal services [8]. The case studies from Nigeria [9] and Tanzania [10] consider attempts to expand health insurance that had been initiated for civil servants. This series of papers adds to the accumulating evidence on the many issues that have to be addressed for the attainment of UHC and to the research agenda on further efforts in this direction, both of which are key concerns of the WHR 2013.

At the same time, a review of the financial burden stemming from non-communicable diseases (NCDs) in low- and middle-income countries was also published in *HARPS* by Kankeu et al. [12]. This review adds further evidence on the need for universal financial protection, with the authors stating that ‘The financial costs of obtaining care also impose insurmountable barriers to access for some people, which illustrates the urgency of improving financial risk protection in health in [low- and middle-income country] settings and ensuring that NCDs are taken into account in these systems’ [12], p. 1. The authors also set out an agenda for further research in this field.

### All nations should be producers and consumers of research

The evidence from the papers described above also illustrates the relevance of the second message from WHR 2013, i.e., the need for all nations to be involved in health research since many questions about UHC ‘require local answers’ [1], p. xiii. Research should be conducted not only in academic centres but also in the public health programmes that are close to the supply of health services. It argues that by strengthening health research systems, ‘countries will be able to capitalize more effectively on the supply of ideas, using formal research methods to turn them into useful products and strategies for better health’ [1], p. xiii.

The report traces the efforts made to boost the involvement of all countries in research, starting with the 1990 report of the Commission on Health Research for Development [13], which recommended that all countries undertake and support essential national health research. A 20^th^ anniversary symposium held to assess progress made in strengthening essential national health research capacity in developing countries and in global research partnerships was described by Frenk and Chen [14]. They noted the progress and concluded that ‘All nations must participate in the advancement and sharing of research-generated knowledge, along with developing the capacity to not just adopt the evidence, but to adapt it to local circumstances’ [14], p. 4.

Countries are always going to rely substantially on evidence produced globally by the research community. This is clearly illustrated by the UK, a country that makes a major contribution to the stock of global health research papers (providing about 10% of the total), but even this is not sufficient to provide more than a minority of the research papers (about 17%) cited on key UK cardiovascular disease guidelines [15]. Therefore, the question becomes one of finding the right balance for each country.

The WHR 2013 describes the recent growth in health research and states that ‘A greater recognition of the value of research for health, society and the economy has added impetus to the upward trend’ [1], p. 31. The report describes various studies showing how the value of research had been demonstrated, but also quotes a review conducted in Iran [16] showing that such studies have been concentrated in high-income countries, a finding confirmed in a later review of research impact studies [17].

A recently published report collates evidence to indicate a further way in which benefits arise from research being conducted widely within each healthcare system [18]. This synthesis provides evidence to support the widely assumed, yet relatively undocumented, view that ‘when clinicians and health-care organisations engage in research there is the likelihood of a positive impact on health-care performance’ [18], p. vi. The review identifies a range of mechanisms through which research engagement might improve healthcare, but again most of the papers included come from high-income western countries.

### Developing national health research systems

As noted above, the third message from the WHR 2013 highlights the importance of adopting a systems approach to the development of health research in all countries [1]. Chapter 4 of the report sets out, in more detail, the functions that an effective health research system needs to fulfil; it must ‘define research questions and priorities; raise funds and develop research staff capacity and infrastructure; establish norms and standards for research practice; and translate research findings into a form that can guide policy’ [1], p. 95. The articles from *HARPS* that are drawn upon in the discussion cover most of these functions, and include an overview by Terry and van der Rijt of WHO’s own research activities [19]. The report uses a priority setting key question checklist from Viergever et al. [20] as a framework around which examples from various priority setting exercises are described. The WHR 2013 also provides examples of many health topics on which there have been priority-setting exercises. There have been few published national priority-setting exercises, although the Brazilian case is highlighted [21], along with a review of how priorities were set in a range of low- and middle-income countries [22]. This showed that country-level priority setting processes differed significantly in terms of the methods used, yet the paper also noted a relative lack of genuine stakeholder engagement.

The WHR 2013 describes the general principles for strengthening health research capacity framed by the ESSENCE on Health Research initiative published by the Special Programme for Research and Training in Tropical Diseases [23]. The report also draws on accounts published in *HARPS* of progress and difficulties with research capacity strengthening in specific countries. These include lessons to be learnt from health research system development efforts in Guinea Bissau, where the challenge is to develop a nationally oriented research stream ‘in addition to the internationally oriented stream, and explore opportunities for mutual reinforcement’ [24], p. 8. In a sophisticated analysis of projects from four African countries, Bates et al. [25] show how the most useful indicators of research capacity can evolve as programmes mature. One key aspect of capacity is the ability to ensure transparency and accountability in research funding. The report describes the standard methods of accounting for research funding used in the UK across research funders [26], as well as recently identified options for mapping global health research investments [27]. The report also draws on a *HARPS* paper showing how the International Centre for Diarrhoeal Disease Research in Bangladesh (ICDDR,B) successfully found solutions to the problems faced by low- and middle-income countries in persuading external donors to contribute to core funding, and to better align their research funding priorities with the priorities of the centre’s strategic plan [28].

The WHR 2013 highlights two general approaches for translating research into policy [1]. These are the SUPPORT tools for evidence informed health policy-making, which were published as a supplement in *HARPS* in 2009 [29], and the Evidence-Informed Policy Networks, illustrated in the report through examples published by Panisset et al. [30]; this paper covers the scaling-up of malaria treatment in Burkina Faso and zinc treatment for childhood diarrhoea in Bangladesh. Another paper cited by the report focuses on an innovative method for mapping the contribution of research to enhance its impact [31]. The chapter ends by describing the research governance of the WHO Eastern Mediterranean region, one of the few published systematic evaluations of research governance [32]. While this paper was not published in *HARPS*, it further illustrates the value of analysing health research at the systems level and illustrates the type of paper we are keen to publish.

### Turning models into practice

The WHR 2013 succeeds in giving powerful examples of the contributions of health research, and in explaining the case for all nations to be producers and consumers of research [1]. It also succeeds in providing many important examples of progress, and obstacles, in performing the key functions involved in building and operating health research systems. Over the last decade, at least, the WHO, building on an approach set out in the Bangkok Conference on Health Research for Development [33], has generated analysis on the functions of a national health research system. In 2003, Pang et al. proposed a list of major functions for a national health research system, which was defined as ‘the people, institutions, and activities whose primary focus is to generate high quality knowledge that can be used to promote, restore, and/or maintain the health status of populations. It can include the mechanisms adopted to encourage the utilization of research’ [34], p. 1.

Beyond assessing progress across the diverse health research system functions, WHR 2013 proposes that there should be an international body to provide guidance on coordination of research. Such a body, the report suggests, ‘must represent the views of all concerned – researchers, funding agencies, private companies, and civil society and their representative governments in the countries concerned’ [1], p. 136. Not only is this undoubtedly a sensible suggestion in relation to a coordinating body, it can also be argued that in building a successful national health research system it is vitally important explicitly to consider the needs of all stakeholders. A wide-ranging analysis of the development of the English health research system published in *HARPS* in 2010 [35] provides a combined stakeholder and historical analysis, showing how the needs of various actors were met in a series of reforms. The relevance of considering stakeholder perspectives when building national health research systems is illustrated by several aspects of the English health research system: it is now fairly comprehensively addressing the range of functions identified, as shown by the many references to that system in the WHR 2013, and it is striving to address the needs of the healthcare system.

For a full understanding of how the English health research system comes to be in a position to perform so many functions, it is desirable to consider how different stakeholders were successful in getting their concerns addressed in the series of reforms. An early set of reforms, the Rothschild reforms of the 1970s, primarily set out to meet the needs of policymakers in the government’s health department. The NHS R&D/Peckham reforms from the early 1990s aimed to ensure that the research system met the needs of the health service. Subsequently, a more comprehensive strategy was proposed in 2005 by Dame Sally Davies, the Director of R&D, in the *Best Research for Best Health* document [36]. These reforms (and perhaps it is now time to call them the Davies reforms) built on the previous efforts and set out to meet the needs of multiple stakeholders, including patients and medical academics. Various reports in the UK had identified increased pressure on medical academics, and called for actions to be taken to boost their role and address the problem of potentially declining numbers [37]. Thus, a research capacity issue was framed in terms of the needs of the relevant stakeholders, and action was taken. Finally, the needs of diverse stakeholders, including industry and the private sector, were addressed in the Cooksey Review [38] that ran in parallel with the implementation of the *Best Research for Best Health* document [36]. The English health research system continues to develop and attempt to meet the needs of a range of stakeholders, for example, the Research Design Service helps healthcare professionals to develop research proposals that meet the needs of the healthcare system [39].

This discussion suggests that those in a position to help build health research systems should undertake stakeholder analyses in parallel with planning the range of crucial functions identified in WHR 2013. This proposal, stimulated by the WHR 2013, recognises the report is a major step forward in promoting health research, and health research systems, with the aim of improving the prospects of achieving UHC. The WHO, and the lead authors, deserve considerable praise for skilfully amassing and analysing so much data and so many perspectives on this issue, which is of importance for the well-being of millions of people.

## Abbreviations

HARPS: Health Research Policy and Systems; NCDs: Non-communicable diseases; UCH: Universal health coverage; WHO: World Health Organization; WHR: World Health Report.

## Competing interests

SH and MG-B are co-editors of *Health Research Policy and Systems* and both receive funding from a range of organisations with an interest in health research.
